# Effect of Sodium Benzoate Preservative on Micronucleus Induction, Chromosome Break, and Ala40Thr Superoxide Dismutase Gene Mutation in Lymphocytes

**DOI:** 10.1155/2015/103512

**Published:** 2015-02-17

**Authors:** Malinee Pongsavee

**Affiliations:** Department of Medical Technology, Faculty of Allied Health Sciences, Thammasat University, Rangsit Campus, Pathum Thani 12121, Thailand

## Abstract

Sodium benzoate is food preservative that inhibits microbial growth. The effects of sodium benzoate preservative on micronucleus induction, chromosome break, and Ala40Thr superoxide dismutase gene mutation in lymphocytes were studied. Sodium benzoate concentrations of 0.5, 1.0, 1.5, and 2.0 mg/mL were treated in lymphocyte cell line for 24 and 48 hrs, respectively. Micronucleus test, standard chromosome culture technique, PCR, and automated sequencing technique were done to detect micronucleus, chromosome break, and gene mutation. The results showed that, at 24- and 48-hour. incubation time, sodium benzoate concentrations of 1.0, 1.5, and 2.0 mg/mL increased micronucleus formation when comparing with the control group (*P* < 0.05). At 24- and 48-hour. incubation time, sodium benzoate concentrations of 2.0 mg/mL increased chromosome break when comparing with the control group (*P* < 0.05). Sodium benzoate did not cause Ala40Thr (GCG→ACG) in superoxide dismutase gene. Sodium benzoate had the mutagenic and cytotoxic toxicity in lymphocytes caused by micronucleus formation and chromosome break.

## 1. Introduction

Preservatives are the substances added to food to prevent decompositions by microbial growth or undesirable chemical changes. There are many preservatives which are commonly used in food industries including benzoate group, which is used as bacteriostatic and fungistatic in acidic food and drink such as vinegar, carbonated drinks, jams, fruit juice, and condiments. Sodium benzoate is commonly used in worldwide food. Nowadays food and drink consumption involve with these preservatives, due to almost products even fresh or dried food are always added preservatives to extend lifespan. Food and Drug Administration (FDA) regulates the amount of food additives allowable in foods or other goods to help ensure safety and reduce the possibility of overconsumption. For using benzoate group such as sodium benzoate and potassium benzoate in dairy products such as ice cream, pudding, and yoghurt, FDA allows using sodium benzoate at 300 mg/1 kg. As the result of long term intake even though it is small amount, the preservatives may cause harm to consumers within some sickness and in chromosomes level. The following adverse effects of food preservatives are nausea, vomiting, diarrhea, rhinitis, bronchospasm, migraine, anaphylaxis, and hyperactivity in children [1].

Micronucleus test (MN) is the test system for the detection of chemicals which induces the formation of micronuclei in the cytoplasm of binucleated cells. These micronuclei may originate from chromosome fragments or whole chromosomes which are unable to migrate with the rest of the chromosomes during the anaphase of cell division [2]. Due to the toxicity of sodium benzoate to human, the purpose of this study was to evaluate the potential genotoxic effects of sodium benzoate which was one of the food preservatives on human lymphocytes.

Extracellular superoxide dismutase (EC-SOD) is an enzyme that in humans is encoded by the* SOD3* gene. This gene encodes a member of the superoxide dismutase (SOD) protein family. SODs are antioxidant enzymes that catalyze the dismutation of two superoxide radicals into hydrogen peroxide and oxygen. The product of this gene is thought to protect the brain, lungs, and other tissues from oxidative stress. The protein is secreted into the extracellular space and forms a glycosylated homotetramer that is anchored to the extracellular matrix (ECM) and cell surfaces through an interaction with heparan sulfate, proteoglycan, and collagen. A fraction of the protein is cleaved near the C-terminus before secretion to generate circulating tetramers that do not interact with the ECM [3].

Oxidative stress has been implicated in pancreatic *β*-cell damage, insulin resistance, and vascular function in diabetic patients. The missense mutation Ala40Thr (GCG→ACG) of* EC-SOD* gene is associated with insulin resistance and the susceptibility to type 2 diabetes [4]. The toxicity of sodium benzoate involved in type 2 diabetes on* EC-SOD* mutation was investigated in this study.

## 2. Materials and Methods

The study was carried out using human lymphocyte cell line (ATCC PCS-800-013).

### 2.1. Study of the Effects of Sodium Benzoate on Micronucleus Test and Chromosome Breakage

Human lymphocyte cell line (ATCC PCS-800-013) was cultured in RPMI medium containing 10% fetal bovine serum, antibiotics, and phytohemagglutinin M. The human lymphocyte cultures were incubated in 5% CO_2_ incubator at 37°C. The concentrations of sodium benzoate were 0.5, 1.0, 1.5, and 2.0 mg/mL and the incubation times for treating lymphocytes were 24 and 48 hrs, respectively, for micronucleus test and chromosome study in this study. For micronucleus test, micronuclei preparation was performed according to the procedures of Fenech [5] and Palus et al. [6]. For chromosome study, standard human lymphocyte culture for chromosome study was performed and stained the chromosomes with Giemsa [7]. Micronuclei and chromosome breakage were observed under microscope.

### 2.2. Study of the Effect of Sodium Benzoate Toxicity on Ala40Thr Superoxide Dismutase Gene Mutation in Lymphocytes

Lymphocytes were treated with sodium benzoate concentration of 2.0 mg/mL for 48 hrs. After that, DNA was extracted from treated lymphocytes with DNA extraction kit followed by PCR [4] and automated DNA sequencing.

### 2.3. Statistical Analysis

The correlations among micronucleus formations, the concentrations of sodium benzoate, and the incubation times were tested by ANOVA at *P* < 0.05 level.

## 3. Results and Discussion

### 3.1. Study of the Effects of Sodium Benzoate on Micronucleus Test and Chromosome Breakage

**Figure 1:**
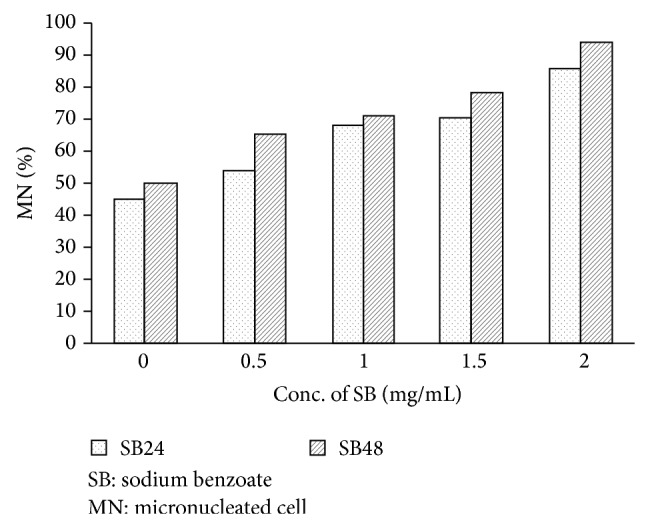
Percent of micronucleated cell in lymphocytes for four sodium benzoate concentrations at 24- and 48-hour incubation time.

**Figure 2:**
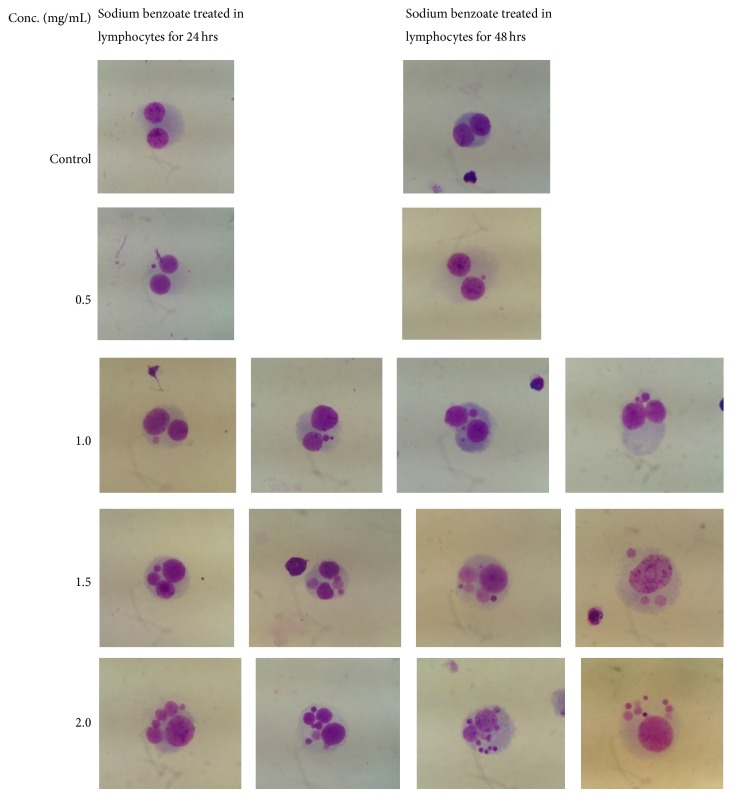
Micronuclei in lymphocytes for four sodium benzoate concentrations at 24 and 48 hrs.

For micronucleus formation, the results showed that the micronucleated cells were increased when the concentration of sodium benzoate increased. At 24- and 48-hour incubation times, sodium benzoate concentrations of 1.0, 1.5, and 2.0 mg/mL increased the micronucleated cells significantly when compared with the control group (*P* < 0.05) but sodium benzoate concentration of 0.5 mg/mL did not increase the micronucleated cells significantly when compared with the control group (*P* > 0.05) (Table 1 and Figure 1). Micronucleated cells in each sodium benzoate concentration were shown in Figure 2.

**Figure 3:**
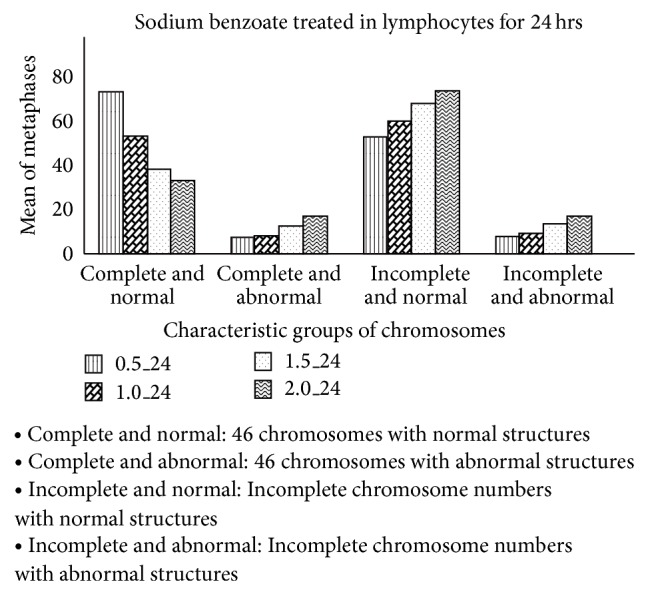
Mean of the numbers of normal and abnormal chromosomes in lymphocytes treated with sodium benzoate at 24-hour incubation time.

**Figure 4:**
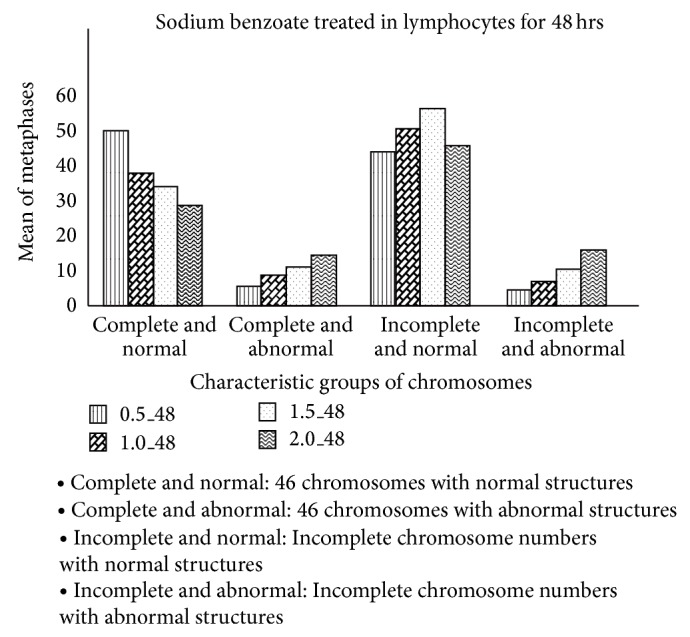
Mean of the numbers of normal and abnormal chromosomes in lymphocytes treated with sodium benzoate at 48-hour incubation time.

**Figure 5:**
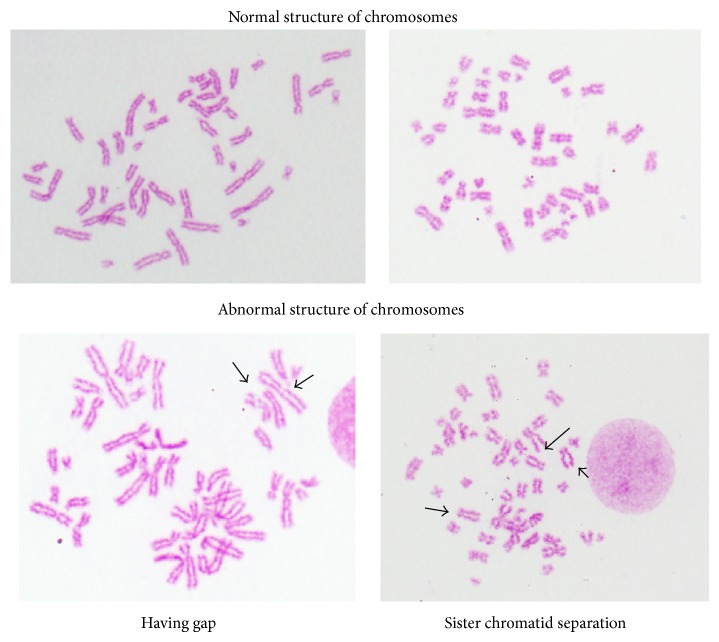
Normal chromosomes and abnormal chromosomes with gap and sister chromatid separation in chromosomes.

For chromosome breakage, at 24- and 48-hour incubation time, sodium benzoate concentration of 2.0 mg/mL caused sister chromatid separation and gap in chromosomes when compared with the control group (*P* < 0.05) but sodium benzoate concentrations of 0.5, 1.0, and 1.5 mg/mL did not cause sister chromatid separation and gap in chromosomes when compared with the control group (*P* > 0.05) (Figures 3 and 4). Sister chromatid separation and gap in chromosomes were shown in Figure 5.

### 3.2. Study of the Effect of Sodium Benzoate Toxicity on Ala40Thr Superoxide Dismutase Gene Mutation in Lymphocytes

The missense mutation Ala40Thr of superoxide dismutase gene in lymphocytes was not found in this study. Thus, sodium benzoate toxicity was not involved in this mutation and type 2 diabetes occurrence.

Sodium benzoate is a preservative. As a food additive, sodium benzoate has the E number E211. It is bacteriostatic and fungistatic under acidic conditions. The mechanism starts with the absorption of benzoic acid into the cell. If the intracellular pH falls to 5 or lower, the anaerobic fermentation of glucose through phosphofructokinase decreases sharply which inhibits the growth and survival of microorganisms that cause food spoilage.

Ishidate et al. [8] reported that sodium benzoate caused chromosome aberrations in Chinese hamster fibroblast cell line. In an in vitro study, ornithine transcarbamylase and tyrosine aminotransferase, marker enzymes in the mitochondria and cytosol of rat liver hepatocytes, were clearly suppressed by sodium benzoate at concentration in excess of 500 *μ*g/mL and inhibited DNA synthesis with 100 *μ*g/mL. Oyanagi et al. [9] and Mpountoukas et al. [10] studied the genotoxic, cytostatic, and cytotoxic potential of sodium benzoate in human peripheral blood cells in vitro. They reported sodium benzoate concentrations of 2.0, 0.2, and 0.02 mM caused no cytostatic activity detected and 4 and 8 mM caused weak cytostaticity. They concluded that the preservatives can be nongenotoxic at low concentrations. Türkoğlu [11] and Zengin et al. [12] studied the effects of sodium benzoate of 20 to 100 ppm for 5, 10, and 20 hrs on root tips of* Allium cepa*. As compared with control, mitotic index values were decreased and chromosome aberrations (anaphase bridges, C-mitosis, micronuclei, lagging, stickiness, breaks, and unequal distribution) increased with increasing concentrations and the longer period of treatment. The toxicity of sodium benzoate did not cause missense mutation Ala40Thr of superoxide dismutase gene in lymphocytes and was not associated with type 2 diabetes occurrence.

## 4. Conclusions

According to our study, it might be the possible reasons of the sodium benzoate, affected DNA damage. The results observed in this study obtain the progress knowledge to prevent diseases or decrease the changes of human chromosomes caused by consumption of food additives and insist on more extensive safety assessment of food preservatives to activate the government departments responsible for public health to be concerned about disadvantages of food additives. However, studying in vivo is required to reach a whole decision about preservative genotoxicity and sodium benzoate mechanism of action on the DNA damage in human.

## Figures and Tables

**Table 1:** Effect of four sodium benzoate concentrations at 24 and 48 hrs on micronucleus formation in lymphocytes.

Treated substance	Treatment		MN ± SE (%)
	Period (hrs)	Conc. (mg/mL)	
Control	24	0	42.88 ± 2.88
SB	24	0.5	51.52 ± 1.62
	24	1.0	66.67 ± 2.10^*^
	24	1.5	69.23 ± 1.36^*^
	24	2.0	86.67 ± 1.77^*^

Control	48	0	50.00 ± 2.88
SB	48	0.5	63.64 ± 1.01^*^
	48	1.0	69.23 ± 1.05^*^
	48	1.5	78.57 ± 1.17^*^
	48	2.0	93.64 ± 1.42^*^

^*^Significantly different from the control (*P* < 0.05).

SB: sodium benzoate.

MN: micronucleated cell.
